# Supplementary material for: BIGFAM - variance components analysis from relatives without genotype
Source: Nat Commun. 2025 Jul 1;16:5476. doi: 10.1038/s41467-025-60502-0 (PMC12217023; doi:10.1038/s41467-025-60502-0)
Supplement: Supplementary file 1 — Supplementary Information [file 41467_2025_60502_MOESM1_ESM.pdf]

Title

BIGFAM - variance components analysis from relatives without genotype

Supplementary Methods

Correlation of genotype of X chromosome for each relationship pair

The core of genotype-free heritability estimation lies in the comparison of phenotypic correlation to the genotype correlation inferred from the relationship. Because of the relation-specific inheritance pattern of the X chromosome, the correlation of the genotype of the X chromosome for a specific relationship pair can be different from that of the autosome. Here, we calculated the correlation of the X chromosome genotype depending on the family relationship by accounting for the unique inheritance pattern of the X chromosome.

For the jth variant on the X chromosome with minor allele frequency (MAF) p_j, the standardized genotype (x) can be expressed as

$$x_{j,k} = \frac{g_{j,k} - E[g_{j,k}]}{SD[g_{j,k}]}$$

where j is the index of the variant, k is the index of individual, and $g_{j,k}$ is the dosage of the genotype. Depending on the sex of k, $g_{j,k}$ is coded as $0,1$ for males and $0,1,2$ for females. $E[g_{j,k}]$ is the expectation of $g_{j,k}$ which is p_j for males and $2p_j$ for females. $SD[g_{j,k}]$ is the standard deviation of $g_{j,k}$ which is $\sqrt{p_j(1-p_j)}$ for males and $\sqrt{2p_j(1-p_j)}$ for females.

For the father-son relationship, the correlation of the genotype of a variant j is expressed as follows,

$$E[X_{j,father} \cdot x_{j,son}] = E[\frac{g_{j,father} - p_j}{\sqrt{p_j(1-p_j)}} \cdot \frac{g_{j,son} - p_j}{\sqrt{p_j(1-p_j)}}].$$

Since a son does not inherit an X chromosome from his father, $g_{j,father}$ and $g_{j,son}$ can be regarded as independent random variables. Consequently, the correlation between the genotype of father and son is zero. Therefore, the correlation between the genotype of the father and son becomes:

$$E[x_{j,father} \cdot x_{j,son}] = 0.$$

For the mother-son relationship, the correlation of the genotype of a variant j is expressed as

$$E[X_{j,mother} \cdot x_{j,son}] = E[\frac{g_{j,mother} - 2p_j}{\sqrt{2p_j(1-p_j)}} \cdot \frac{g_{j,son} - p_j}{\sqrt{p_j(1-p_j)}}].$$

If we separate the genotype into haplotype, the equation can be expressed as follows:

$$E[X_{j,mother} \cdot x_{j,son}] = E[\frac{a^1_{j,mother} + a^2_{j,mother} - 2p_j}{\sqrt{2p_j(1-p_j)}} \cdot \frac{a^1_{j,son} - p_j}{\sqrt{p_j(1-p_j)}}]$$

where a^1_j and a^2_j are the two alleles possessed by the mother. Without loss of generality, we assume that the son inherited a^1_j from the mother. Since $a^1_{j,son}$ is identical with $a^1_{j,mother}$ and is independent of $a^2_{j,mother}$, the correlation between the genotype of the mother and son becomes:

$$E[x_{j,mother} \cdot x_{j,son}] = 1/\sqrt{2}.$$

In the same way, the genetic correlation on the X chromosome of l-relationship ($r_{X,l}$) can be easily derived in first-degree relatives. For the second and more degree-of-relatedness, $r_{X,l}$ can also be easily derived recursively using $r_{X,l}$ in first-degree relatives.

Assortative mating

Under the assortative mating, the heritability estimated from parent-offspring regression can be inflated due to the genetic resemblance between close relatives[[1]]. Herein, we briefly explained about how the assortative mating can affect to the heritability estimation computed in traditional additive genetic model.

The traditional additive genetic model describes the relationship between phenotype and genotype as $y = x^T\beta + \epsilon$. Here, y is a standardized phenotype value, x is a $M \times 1$ standardized genotype vector where M is the number of causal variants, and β is an $M \times 1$ effect size vector which follows normal distribution $N(0, \frac{h^2}{M})$. h^2 is the narrow-sense heritability, and ϵ is an environmental effect value which follows $N(0,1-h^2)$. Suppose that we regress the offspring's phenotype value on parent's phenotype value with regression coefficient λ ($y_{\text{offspring}} = \lambda \cdot y_{\text{parent}}$).

By the standard regression formula,

$$E[\hat{\lambda}] = E[\frac{Cov(y_o, y_p)}{Var(y_p)}] = \sigma(y_o, y_p)$$

where y_o and y_p are the standardized phenotype of offspring and parent respectively. $\sigma(y_o, y_p)$ denotes population covariance between y_o and y_p. Under the additive genetic model, $\sigma(y_o, y_p)$ can be represented as

$$\sigma(x_o^T\beta + e_o,\ x_p^T\beta + e_p) = \sum_{i=1}^{M} E\,[(x_{i,o} \cdot x_{i,p} \cdot \beta_i \cdot \beta_i + x_{i,o} \cdot \beta_i \sum_{j \neq i}^{M} x_{j,p} \cdot \beta_j)] = \frac{1 + r_{AM}}{2} \cdot h^2$$

where $x_{i,o}$ and $x_{i,p}$ is the ith allele of offspring and parents, respectively. β_i is the effect size of the ith allele and r_{AM} the correlation induced by assortative mating.

Without assortative mating, the r_{AM} is assumed to be zero. However, under assortative mating, r_{AM} is greater than zero[[2]][[3]]. In this situation, the variance components by genetic effect (V_g) are overestimated compared to the true heritability (h^2). Nevertheless, our model can still effectively partition the variance components by genetic and shared environmental effects. This is because, even under assortative mating, the variance component by genetic effect in the FR-reg coefficient ($\hat{\lambda}$) still decays as a factor of 2.

Y chromosome

The Y chromosome, along with the X chromosome, presents distinctive inheritance patterns that differ from autosomes. Unlike the X chromosome, the Y chromosome is exclusively passed down from father to son through male lineages.

However, the Y chromosome’s contribution to overall heritability estimates tends to be relatively minor. This is primarily due to its smaller size and lower gene content compared to other chromosomes. For instance, the Y chromosome spans approximately 57 million base pairs (bp) and contains only around 200 genes, whereas the X chromosome, with a length of 156 million bp, contains over 1400 genes. This limited gene content restricts its contribution to the total genetic variation underlying complex traits[4].

If the Y chromosome does exhibit significant heritability, its inclusion in heritability estimation could potentially introduce bias. Nonetheless, the impact of such bias may diminish when analyzing multiple relatives, as the averaging effect across relatives can help mitigate these biases.

Consequently, while the Y chromosome is crucial for male-specific inheritance, its overall influence on heritability estimates for complex traits is expected to be modest.

Various approaches for estimating variance components by genetic effects

To compare genetic variance components estimated from BIGFAM with other methods, we categorized existing approaches into three broad categories based on their data requirements. The first approach relies solely on GWAS summary statistics (population-level genetic association data), including methods such as LDSC and LDpred. The second approach utilizes

individual-level genotype data from relatives, as implemented in methods like Haseman-Elston (HE) regression, REML-based big-K small-K (bKsK), and Relatedness Disequilibrium Regression (RDR). The third approach, which includes BIGFAM and Structural Equation Model (SEM), operates without genetic data and instead uses only family relationships to estimate genetic effects through expected inheritance patterns.

For methods using GWAS summary statistics (LDSC, LDpred), we utilized UK Biobank summary statistics. For methods analyzing relatives (both with and without genotype data), we included first- to third-degree relatives in our analysis. The sample comprised 81,326 relative pairs (123,418 individuals) from UK Biobank and 38,006 relative pairs (18,006 individuals) from GS:SFHS.

LDSC

LD Score Regression (LDSC)[5] is a method for estimating heritability using GWAS summary statistics (so called LDSC heritability). This approach leverages the relationship between the linkage disequilibrium (LD) score of each SNP and its association test statistic. Specifically, the expected chi-squared statistic (χ^2) for a SNP can be expressed as:

$$E(\chi^2) = 1 + N \cdot h^2 \cdot l^2$$

where N is the sample size, h^2 is the heritability, and l represents the LD Score. The intuition behind this method is that variants in regions of high LD are expected to show stronger associations if they tag true causal effects.

LDpred

LDpred2[6] also a method for estimating heritability using GWAS summary statistics (so called LDpred2 heritability). By extending the LDSC, this method addresses potential biases in complex traits (e.g., oligogenic phenotypes) where only a small fraction of variants are truly

causal. The method combines Bayesian priors to model the sparsity of causal effects with LD information. The heritability estimate in LDpred2-auto can be expressed as:

$$h^2 = \frac{\sum_j \hat{\beta}_j^2 \cdot N}{\sum_j l_j}$$

where $\hat{\beta}_j$ represents the observed effect size of SNP j, N represents the sample size, and l_j is the LD score for SNP j. LDpred2-auto iteratively refines this estimate by jointly modeling the heritability (h^2) and the proportion of causal variants (p), making it particularly robust for traits where only a small subset of variants has true effects.

Haseman-Elston (HE) regression

Haseman-Elston regression estimates heritability using genetic relatedness between pairs of relatives computed from SNP data (so called HE heritability). This method provides two conceptually different approaches for analyzing the relationship between phenotypic similarity and genetic relatedness.

The first approach utilizes the squared difference of phenotypes between pairs of individuals (HE-SD). This method assumes larger phenotypic differences to correspond to lower genetic relatedness:

$$(y_i - y_j)^2 \sim 2 \cdot v_p - 2r_{ij} \cdot v_g$$

where y_i and y_j are the phenotypes of related individuals i and j, v_p represents the total phenotypic variance, v_g denotes the variance component by genetic effect (HE-SD heritability), and r_{ij} indicates the genetic relatedness between individuals i and j.

The second approach employs the cross product of phenotypes (HE-CP). This method assumes larger phenotypic cross products to correspond to higher genetic relatedness:

$$(y_i \cdot y_j) \sim r_{ij} \cdot v_g + 1_{i=j} \cdot v_e$$

where v_g denotes the variance component by genetic effect (HE-CP heritability), v_e denotes the variance component by environmental effect, and $1_{i=j}$ represents indicator vector which is 1 if $i = j$ otherwise 0.

In our analysis, we implemented both approaches using GCTA software's –HEreg command for HE regression, and –make-grm command for computing the genetic relatedness ($r_{i,j}$)[[7]][[8]].

Big-K small-K

REML-based methods estimate heritability using genetic relatedness computed from SNP data of relatives. However, when applied directly to relatives, estimates from REML can be biased due to shared environmental effects[[8]].

To address this, the big-K small-K method[[9]] was developed. This method divides genetic relatedness into two matrices using a cutoff value (0.05) and jointly fits them using REML. This approach enables estimation of both SNP heritability and pedigree heritability through the following model:

$$\text{Cov}(Y) = h_{snp}^2 \cdot K + (h_{ped}^2 - h_{snp}^2) \cdot K_t + (1 - h_{ped}^2) \cdot I$$

where h_{snp}^2 represents the SNP heritability, h_{ped}^2 is the pedigree heritability, K is the genetic relatedness matrix from relatives (big-K), K_t is an additional genetic relatedness matrix derived from K by setting off-diagonal elements less than 0.05 to zero (small-K matrix), and I is the identity matrix.

Relatedness Disequilibrium Regression

Relatedness Disequilibrium Regression (RDR)[[10]] estimates heritability using both individual-level genotype data and familial relationships (so called RDR heritability). This method analyzes

how genetic relatedness between relatives deviates from expected patterns, referred to as relatedness disequilibrium.

RDR considers multiple genetic relatedness matrices simultaneously: relatedness between individuals (R), between their parents (R_{par}), and between individuals and others' parents ($R_{o,\text{par}}$). The RDR covariance model is expressed as:

$$\text{Cov}(Y) = v_g \cdot R + v_{e\sim g} \cdot R_{\text{par}} + c_{g,e} \cdot R_{o,\text{par}} + \sigma^2 \cdot I$$

where Y represents the phenotype vector, v_g represents RDR heritablity, $v_{e\sim g}$ represents environmental variance correlated with parental genotypes, $c_{g,e}$ represents the covariance between direct genetic and environmental effects, and σ^2 is the residual variance. By doing so, RDR provides robust heritability estimates even in the presence of confounding environmental factors.

Structural Equation Model (SEM)

Structural Equation Model (SEM) estimates heritability using only familial relationships without genotype data (so called SEM heritability). This statistical approach estimates variance components by modeling the correlation structure between relatives, decomposing phenotypic correlations between pairs of relatives into genetic and environmental components. By extending SEM on the first to third degree relatives, we construct the basic form of the SEM as follows:

$$Cor(y_i, y_j) \sim v_a \cdot A^* + v_{s_1} \cdot S_1 + v_{s_2} \cdot S_2 + v_{s_3} \cdot S_3$$

where v_a represents the pedigree-based heritability, and v_{s_k} represents the variance component due to shared environmental effects between k-degree relatives. A^* is the genetic relatedness matrix based on expected genetic sharing between relatives (1/2 for first-degree relatives, 1/4

for second-degree relatives, and 1/8 for third-degree relatives). S_k is the shared environment matrix for k-degree relatives, where elements are 1 if the pair are k-degree relatives and 0 otherwise.

REML approach without genotype data

We explored whether REML could be similarly applied using only familial relationships. To this end, we constructed multiple relationship matrices based on pedigree information. First, an pedigree-based additive genetic relatedness matrix ($\mathbf{A}^*$) is constructed with coefficients of $(0.5)^d$ for *d*-degree relative pairs and 0 for unrelated pairs. Next, for each degree-of-relatedness, we construct a shared environmental relatedness matrix ($\mathbf{S_d}$) with coefficients of 1 for *d*-degree relative pairs and 0 for unrelated pairs. With these matrices, the model can be expressed as:

$$\text{Cov}(Y) = V_A \cdot \mathbf{A}^* + \sum_{d}^{D} V_{S_d} \cdot \mathbf{S_d} + V_E \cdot \mathbf{I}$$

where Y represents the phenotype vector, V_A represents the (pedigree) heritability, V_{S_d} represents the variance component due to shared environmental effects between d-degree relatives, V_E represents the residual variance, and D represents the number of degrees-of-relatedness included in the analysis (e.g., $D = 3$ when first, second, and third-degree relatives are used).

However, when we applied this model to our dataset including first to third-degree relatives ($D = 3$), it failed to achieve convergence. In the following sections, we demonstrate why REML-based approaches without genotype data show fundamental numerical stability issues when estimating variance components from relatives.

Numerical Instability in Multiple GRM Estimation

When we applied REML on above model, we observed severe fluctuations in likelihood values and variance component estimates across iterations, ultimately failing to achieve convergence. To investigate the source of this instability, we examined the estimation process in detail and found extremely high condition numbers ($10^{16} - 10^{17}$) in the average information (AI) matrix, which is inverted during the REML framework. These large condition numbers indicate a substantial ratio between the largest and smallest eigenvalues of the matrix, leading to severe numerical instability during matrix inversion.

While various factors can lead to high condition numbers in matrix operations, in our model, the following three factors are possible to be the main contributors to numerical instability:

1. **Number of Parameters to Estimate**: The model requires the estimation of five parameters ($V_A, V_{S_1}, V_{S_2}, V_{S_3}, V_E$). As the number of parameters increases, the AI matrix becomes larger and more complex, making it more susceptible to numerical instability during inversion.
2. **Matrix Sparsity**: The pedigree-based genetic relationship matrix ($\mathbf{A}^*$) is inherently sparse, as coefficients are only non-zero between related individuals. For instance, with N pairs of relatives, only N out of $\frac{N(N-1)}{2}$ off-diagonal elements in $\mathbf{A}^*$ would contain non-zero values.
3. **Multicollinearity**: There exists strong collinearity among the relatedness matrices. For instance, knowing that a pair (i,j) has $(\mathbf{S_1})_{i,j} = 1$, we can deterministically know that other $(\mathbf{S_d})_{i,j}$'s are 0 and $(\mathbf{A}^*)_{i,j}$ is $1/2$.

To further investigate which factor contributes most to the instability, we conducted experiments with two different models: (1) reduce the number of parameters by using a single shared environmental matrix, and (2) mitigate matrix sparsity and multicollinearity by substituting the pedigree-based genetic relationship matrix ($\mathbf{A}^*$) with SNP-based additive genetic relationship matrix ($\mathbf{A}$).

Using a single shared environmental matrix

To address the first issue of having too many parameters to estimate, we simplified the model by introducing a single shared environmental matrix with a shared environmental decaying factor (w_S). This shared environmental matrix is constructed with coefficients of 1 for first-degree relatives, $1/w_S$ for second-degree relatives, and $1/w_S^2$ for third-degree relatives. By doing this, we can reduce the number of parameters to estimate from five to three (v_a, v_s, and v_e).

$$\mathrm{Cov}(Y) = V_A \cdot \mathbf{A}^* + V_S \cdot \mathbf{S}_{\mathbf{w_S}} + v_E \cdot \mathbf{I}$$

With this modification, the numerical instability issue was resolved, showing stable monotonic convergence of the likelihood function. The condition number of the AI matrix was substantially reduced from the previous extremely large value (10^16 - 10^17) to ~500.

However, this approach presents significant limitations. The choice of w_S is arbitrary as existing REML-based methods do not optimize this parameter. Moreover, the estimated variance components vary substantially depending on the choice of w_S. For instance, in our analysis of Urate, (V_A, V_S) was estimated as $(0.41{,}0.03)$, $(0.50{,}0.00)$, and $(0.46{,}0.02)$ for w_S values of 1.1, 4.0, and 100, respectively. In summary, while using a single shared environmental matrix resolves the numerical instability issue, the strong dependence of the estimates on the arbitrary choice of w_S makes it an unreliable method for variance component estimation.

Substituting the pedigree-based genetic relationship matrix with SNP-based additive genetic relationship matrix

To address the matrix sparsity and multicollinearity, we replaced the pedigree-based additive genetic matrix ($\mathbf{A}^*$) with a SNP-based additive genetic relationship matrix ($\mathbf{A}$). This substitution directly addresses the sparsity issue, as the SNP-based matrix contains non-zero values for all pairs of individuals. Moreover, this change also reduces multicollinearity among matrices, as the relationship between $\mathbf{A}$ and environmental matrices ($\mathbf{S_d}$) becomes probabilistic rather than deterministic. Specifically, for a pair of individuals (i, j) where $(\mathbf{S_1})_{i,j} = 1$, we know $(\mathbf{A}^*)_{i,j} = 1/2$ deterministically and $(\mathbf{S_k})_{i,j} = 0$ for other k. However, $(\mathbf{A})_{i,j}$ is distributed around $1/2$ with some random variation, weakening the structural dependencies between jointly fitted matrices. Therefore, the model can be expressed as:

$$\text{Cov}(Y) = V_A \cdot \mathbf{A} + V_S \cdot \mathbf{S_{w_S}} + v_E \cdot \mathbf{I}$$

Despite maintaining the same number of parameters to estimate, this modification also resolved the numerical instability issue, showing stable monotonic convergence of the likelihood function. Specifically, the condition number of the AI matrix was reduced to ~200, which is even lower than that of the single shared environmental matrix model (~500). This substantial improvement in numerical stability suggests that using observed genetic relationships rather than expected relationships effectively reduces the structural dependencies among the matrices.

Other approaches using REML without genotype data

Tools such as AsREML support estimating heritability without genotype data using pedigree information (through the *ainverse()* function). This function constructs the inverse of the additive

genetic matrix using pedigree information enabling faster REML computation. With this inverse matrix, AsREML estimates heritability using REML by jointly fitting two matrices: the pedigree-based additive genetic matrix ($\mathbf{A}^*$) and the individual-specific environmental matrix ($\mathbf{I}$)[11]. This model converges well without numerical issues, however, this approach does not account for shared environmental effects. With shared environmental effect, this approach can yield biased estimate. Yang et al.[8] demonstrated that heritability estimates from REML with only additive genetic matrix can be overestimated under the presence of shared environmental effects.

To address this limitation and control for shared environmental effects, several methods have been proposed that apply REML to relative samples, including big-K small-K[9], relatedness disequilibrium regression[10], and GREML-KIN[12]. These methods share a common framework of jointly fitting multiple matrices using REML, typically combining a SNP-based additive genetic relationship matrix with additional environmental matrices. While these methods could theoretically be adapted to use pedigree-based matrices instead of SNP-based ones, such an approach would still face the numerical instability issues we demonstrated earlier due to jointly fitting multiple matrices.

In summary, when applying REML without genotype data, the results were unstable due to the inherent properties of the design matrices (such as multicollinearity, sparsity, and number of parameters). Interestingly, we found that stable estimation was possible without genotype data by reducing the number of parameters through the adaptation of a shared environmental decaying factor (w_S). However, the conventional REML framework cannot determine the appropriate value of w_S, and our analysis showed that the estimated variance components varied substantially depending on the choice of this parameter. BIGFAM addresses this limitation by providing a framework to estimate w_S from relative's phenotype data. Therefore, BIGFAM could serve as a complementary tool to the REML framework by providing appropriate

estimates of w_S, enabling reliable variance component analysis using REML on datasets with multiple degrees of relatives.

Comparison of BIGFAM with HE regression

Both HE regression and BIGFAM share fundamental similarities in their approach. In particular, BIGFAM shares methodological similarities with the cross-product approach of HE regression (HE-CP). These methods model the cross-product of phenotypes between relative pairs as a function of various variance components (additive genetic, shared environment, etc.), and use genetic relatedness information in a regression framework for estimation. However, BIGFAM's key distinction and advantage lies in its ability to reduce the number of parameters to estimate, enabling stable estimation not only of genetic and shared environmental variance components but also of X chromosome effects. This feature proves particularly valuable when analyzing datasets with multiple degrees of relatedness.

Similar to our modification of REML models in the previous section, HE regression can also be adapted to operate without genotype data by substituting SNP-based genetic relatedness (a_{ij}) with pedigree-based genetic relatedness (a_{ij}^*). Here, we compare two approaches of HE regression (HE-SD and HE-CP) with BIGFAM, examining their capabilities and limitations in estimating variance components without genotype data.

Square difference of the phenotypes for pairwise individuals

For the squared difference approach (HE-SD), the model can be modified using pedigree-based genetic relatedness (a_{ij}^*) as:

$$(y_i - y_j)^2 \sim 2 \cdot V_P - 2a_{ij}^* \cdot V_A$$

where y_i and y_j are the phenotypes of individuals i and j respectively, V_P is the total phenotypic variance, a_{ij}^* is the pedigree-based genetic relatedness between individuals i and j, and V_A is the variance component attributed to additive genetic effects. Under the ACE model, this approach can estimate V_A by controlling for shared environmental effects through the difference between phenotypes of relatives. This control is achieved under the assumption that pairs of relatives experience similar shared environmental effects, as these effects are eliminated when taking the difference between their phenotypes.

However, this approach has two major limitations. First, it cannot estimate shared environmental effects as these effects are eliminated in the difference between phenotypes of individuals i and j. In contrast, BIGFAM not only estimates genetic effects but also provides comprehensive information about shared environmental effects and their decay patterns across different degrees of relationships. Second, HE-SD shows significant numerical instability when estimating X chromosome heritability. It is theoretically possible to estimate variance components of the X chromosome (V_X) by incorporating pedigree-based X chromosome genetic relatedness (x_{ij}^*) as follows:

$$(y_i - y_j)^2 \sim 2 \cdot V_P - 2a_{ij}^* \cdot V_A - 2x_{ij}^* \cdot V_X.$$

However, the estimation produces unreliable results in practice. Specifically, excessively large standard errors and biologically implausible estimates where X chromosome variance components highly exceed autosomal variance components. When estimating the V_X, BIGFAM addresses this limitation through a fundamentally different approach: it first removes both shared environmental effects and autosomal genetic components that are common to each degree of relationship, thereby isolating the X chromosome effects. This approach reduces the complexity of the estimation problem to focus solely on X chromosome variance components,

leading to more stable estimates. Furthermore, BIGFAM provides additional numerical stability by using L2 penalty.

To demonstrate this, we analyzed the V_X estimate of BIGFAM and HE-SD for 106 phenotypes in the UK Biobank dataset. The results show that while HE regression appeared to detect more traits with non-zero V_X (34 phenotypes) compared to BIGFAM (22 phenotypes), closer examination reveals that the V_X estimates from HE regression frequently showed unreliable patterns: implausibly large effect sizes and excessively wide confidence intervals. For instance, in the *leg fat-free mass*, HE regression estimated $V_X = 0.692$ (95% CIs: [0.571, 0.812]), suggesting that X chromosome alone explains nearly 70% of the phenotypic variance - a biologically implausible result. In contrast, BIGFAM provided a more reasonable estimate of $V_X = 0.016$ (95% CIs: [0.005, 0.030]).

Even in cases where both methods estimated similar V_X values, for example in *LDL-direct*, BIGFAM's estimate of 0.0041 was significant (95% CIs: [0.0002, 0.0075]), while HE regression's estimate of 0.0514 was non-significant due to wide confidence intervals (95% CIs: [-0.0510, 0.1538]).

Cross-product of the phenotypes for pairwise individuals

For the cross-product approach (HE-CP), the model can be modified using pedigree-based genetic relatedness (a_{ij}^*) as:

$$(y_i \cdot y_j) \sim a_{ij}^* \cdot V_A + 1_{i=j} \cdot V_E$$

where $1_{i=j}$ is an indicator vector that equals 1 if $i = j$ and 0 otherwise.

However, this approach yields biased estimates of genetic effects when shared environmental effects are present. To control for this bias, the model can be further modified to include shared environmental effects:

$$(y_i \cdot y_j) \sim a^*_{ij} \cdot V_A + \sum_{d}^{D} 1_{(i,j)\in S_d} \cdot V_{S_d} + 1_{i=j} \cdot V_E$$

where $1_{(i,j)\in S_d}$ is an indicator vector that equals 1 if (i,j) is a d-degree relative and 0 otherwise.

This modification faces the same convergence issues as the REML model without genotype data discussed earlier. Although the estimation approach differs (REML uses matrix operations with the AI matrix, while this approach vectorizes relatedness matrix components for linear regression), both suffer from multicollinearity, sparsity, and the large number of parameters to estimate. In contrast, BIGFAM takes a fundamentally different approach to overcome these limitations. Instead of attempting to estimate all parameters simultaneously, it employs a two-step procedure: using a slope test to estimate the pattern of shared environmental decay, then estimating variance components and the shared environmental decaying factor in the prediction step. This strategic reduction in the number of parameters to estimate effectively addresses the numerical instability issues when analyzing data with multiple degrees of relatives.

Advantages of BIGFAM's Parameter Reduction Strategy in Variance Component Analysis

Both HE regression and BIGFAM share the fundamental concept of estimating variance components using relative data, but they differ significantly in their detailed methodology and capabilities. While both approaches can be implemented without genotype data, they show distinct characteristics in handling various components of genetic and environmental effects.

HE regression faces several limitations. The squared difference approach (HE-SD) controls for shared environmental effects but consequently cannot estimate these effects, and produces unreliable estimates for X chromosome variance components. The cross-product approach (HE-CP), when modified to estimate shared environmental effects without genotype data,

encounters the same numerical instability issues as REML approaches due to the large number of parameters, multicollinearity, and sparsity in the regression covariates.

BIGFAM addresses these limitations through strategic parameter reduction in two ways. First, for estimating autosomal genetic (V_A) and shared environmental (V_S) components, it introduces a shared environmental decaying factor (w_S). This reduces multiple degree-specific V_S parameters to just two parameters: first-degree V_S and w_S. Second, for X chromosome variance component (V_X) estimation, it first removes both autosomal genetic and shared environmental effects that are common to each degree of relatedness. This isolation allows estimation of V_X using only pedigree-based X chromosome genetic correlation (x_{ij}^*), effectively reducing the problem to a single parameter estimation. Additional numerical stability is achieved through L2 regularization.

This parameter reduction strategy makes BIGFAM particularly valuable for analyzing large-scale family datasets by reducing the relationship or degree specific variance components.

Supplementary Figures

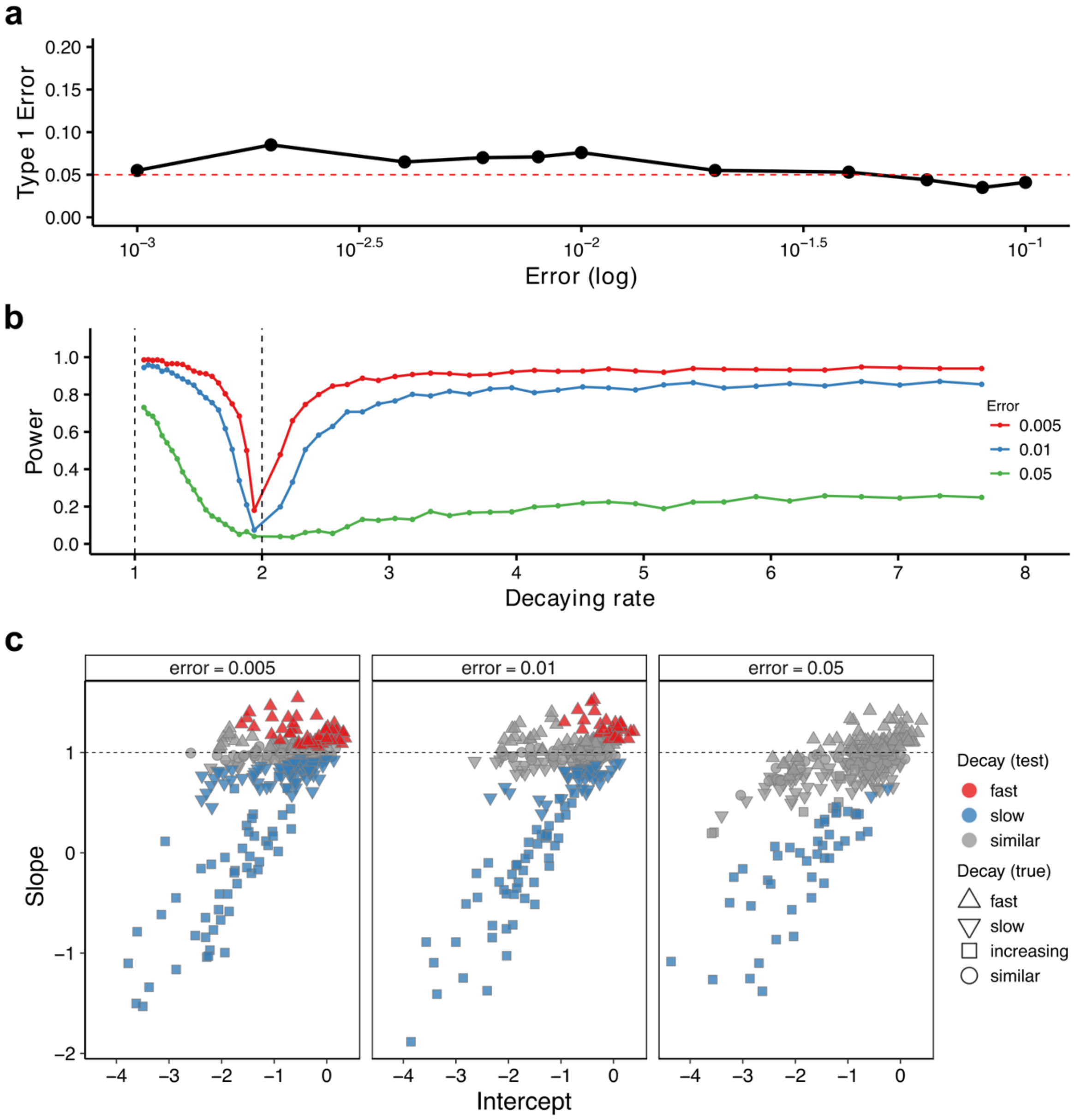

Figure 1. Performance of Slope Test Under Different Error Conditions

a. Assessment of type 1 error rates of the slope test under the null hypothesis ($w_S = 0.5$), across different simulated FR-reg coefficient error levels (ϵ ranging from 0.001 to 0.1 on a logarithmic scale). The dashed red line indicates the expected type 1 error rate (0.05). Type 1 error rates were calculated from n = 1,000 simulated FR-reg coefficients for each error level. The observed type 1 error rate averaged around 5.9%, demonstrating that the slope test is relatively well-calibrated under the null hypothesis.

b. The power of the slope test in identifying scenarios where shared environmental effects decay at different rates compared to genetic effects ($w_S \neq 0.5$). Simulations were conducted across a range of decaying rates, with *slow* decay scenarios ($w_S \in (1,2)$) on the left side of the vertical dashed line and *fast* decay scenarios ($w_S \in (2,8)$) on the right. Power was calculated based on n = 1,000 simulated FR-reg coefficients for each error level. The power is shown for three different simulated FR-reg coefficient error levels ($\epsilon = 0.005,\ 0.01,\ 0.05$), indicating higher power as the true decaying rate diverges further from 2 and as the simulation error decreases

c. Distribution of slope test results under different error conditions. Each panel shows the relationship between intercept and slope from the slope test, with error magnitudes of 0.005, 0.01, and 0.05 (left to right). A random subset on n=300 simulations is shown for clarity. Points are colored by the test result (fast: red, slow: blue, similar: grey) and shaped by the true decay pattern (triangle: fast, inverted triangle: slow, square: increasing, circle: similar). The horizontal dashed line at slope=1 indicates where genetic and shared environmental effects decay at the same rate. FR-reg coefficients were simulated by randomly sampling V_G (0.1-0.8), V_S (0-0.2), and w_S (1/w_S: 0.2-0.9).

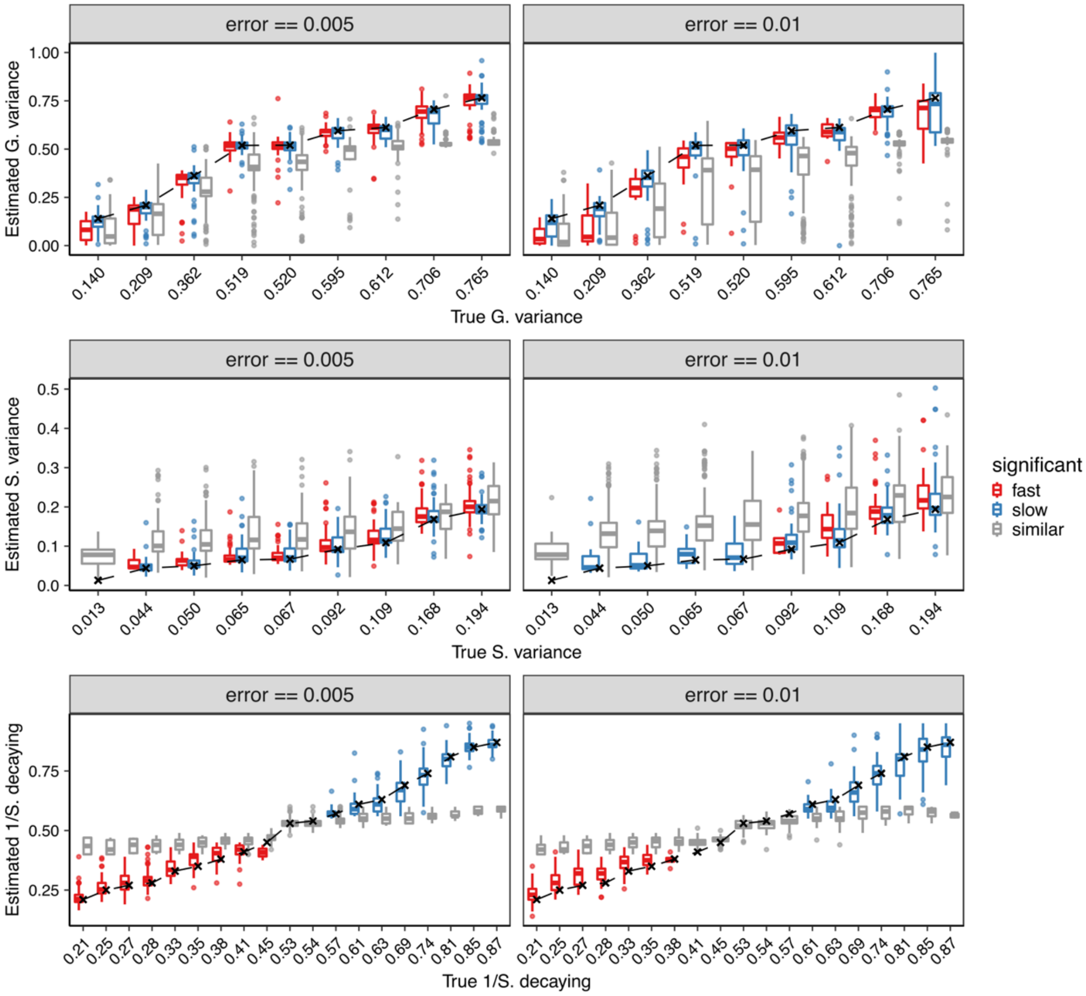

Figure 2. Accuracy of Parameter Estimation Under Different Error Conditions

Comparison between true and estimated parameters under two different error magnitudes (0.005 and 0.01) in the simulated FR-reg coefficients. Each row shows the relationship between true and estimated values for genetic variance (*G.variance*, top), shared environmental variance (*S.variance*, middle), and shared environmental decay rate (1/*S.decaying*, bottom). Box plots represent the distribution of estimated values from 1,000 simulations, with colors indicating the decay pattern classification from the slope test (fast: red, slow: blue, similar: grey). Black lines connect median values across different true parameter values. Parameters were simulated by

randomly sampling genetic variance (V_G: 0.1-0.8), shared environmental variance (V_S: 0-0.2), and shared environmental decay rate ($1/w_S$: 0.2-0.9). Results for error = 0.05 are not shown as they predominantly yielded similar decay patterns, indicating poor discrimination ability at higher error levels. The decay patterns were classified based on the 95% confidence intervals of the slope test: similar (interval includes 1), fast (lower bound > 1), or slow (upper bound < 1).

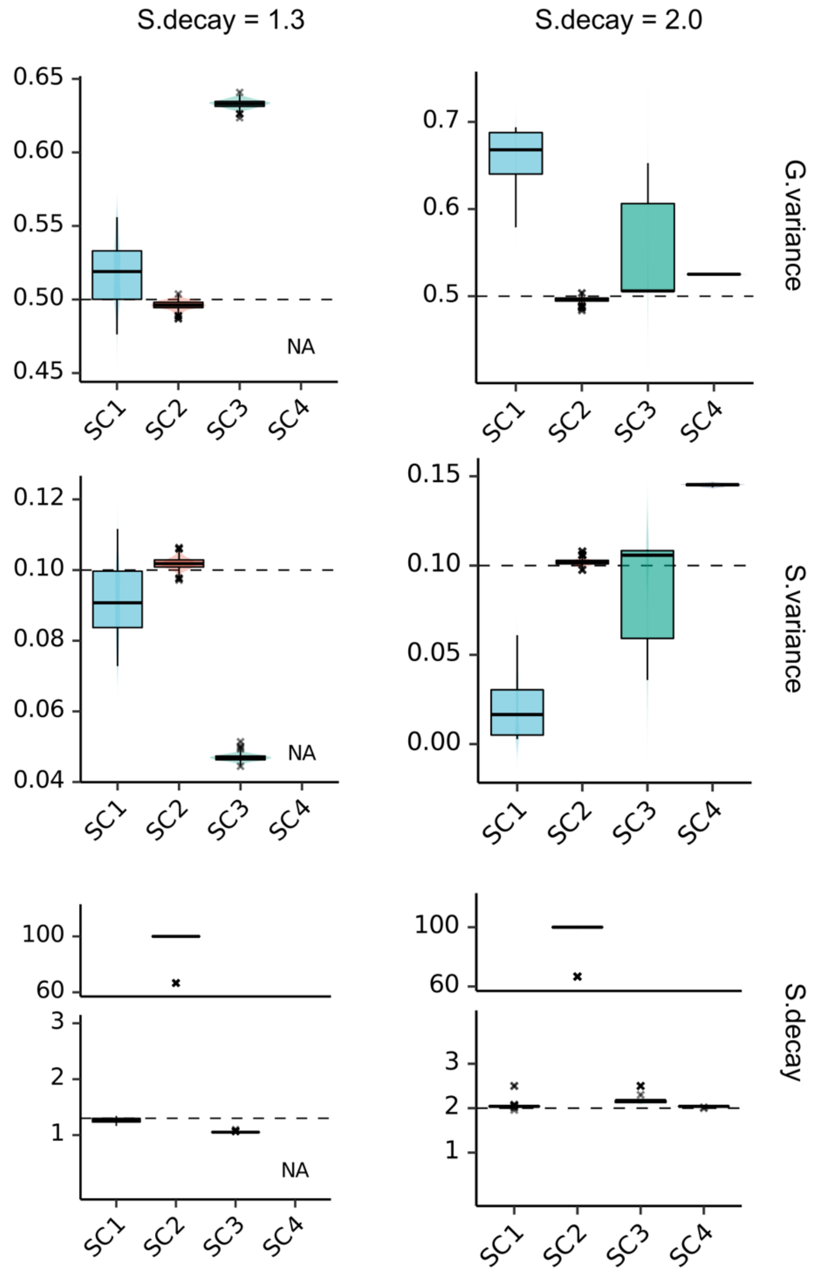

Figure 3. Prediction Performance Under Different Shared Environmental Decay Rates

Evaluation of BIGFAM's prediction performance under slow ($w_S = 1.3$, left) and similar ($w_S = 2.0$, right) decay patterns, complementing the fast decay pattern ($w_S = 5.0$) shown in **Fig.2b**. Four shared environmental scenarios were tested: gradual decay ($SC1$), nuclear-family-specific ($SC2$), maternal-effect ($SC3$), and second-degree-specific ($SC4$). Box plots show the distribution

of the estimated genetic variance (G.variance, top), shared environmental variance (S.variance, middle), and shared environmental decay rate (S.decay, bottom) across 1,000 simulations, performed assuming a standard error of 0.005 for FR-reg coefficients. Boxes represent interquartile ranges (IQR), center lines show medians, and whiskers extend to 1.5×IQR. Dashed lines indicate true values ($V_G = 0.5$, $V_S = 0.1$). Under slow decay ($w_S = 1.3$), maternal effects ($SC3$) lead to less accurate estimates due to inconsistent decay rates between degrees of relatedness. Under similar decay ($w_S = 2.0$), where genetic and shared environmental effects decay at the same rate, the model maintains reasonable performance in most scenarios except $SC4$. "NA" indicates no significant estimates for $SC4$ (i.e., the 95% confidence intervals included zero for all simulations).

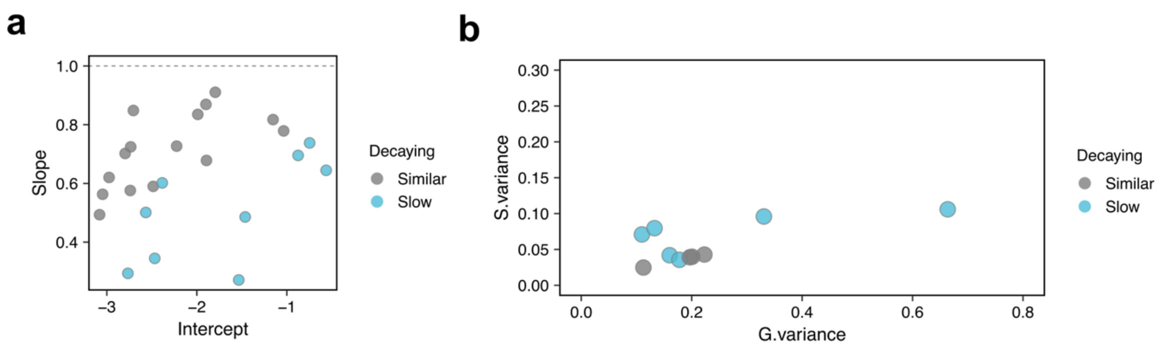

Figure 4. Performance of BIGFAM in Partitioning Genetic and Shared Environmental Effects in GS:SFHS

a. Results of the slope test in GS:SFHS dataset (24 phenotypes). The scatter plot shows the distribution of intercept and slope from slope test. Points are colored based on their shared environmental decay pattern: *slow* (blue, shared environmental effects decay slower than genetic effects), and *similar* (grey, shared environmental effects decay at similar rate with genetic effects). None of fast decay pattern was discovered in this dataset. The horizontal dashed line at slope=1 represents where genetic and shared environmental effects decay at the same rate. **b**. Distribution of estimated variance components in GS:SFHS dataset. The scatter plot shows the relationship between genetic variance (*G.variance*, x-axis) and shared environmental variance (*S.variance*, y-axis). Point colors indicate the decay pattern classification from the slope test as defined in panel a.

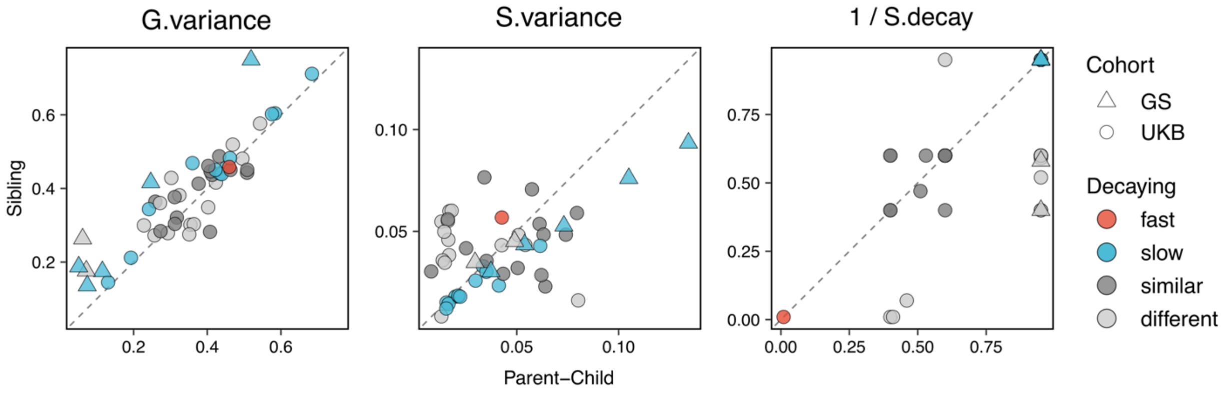

Figure 5. Comparison of Variance Components Between Parent-offspring and Sibling Pairs

Scatter plots comparing variance component estimates derived separately from parent-offspring (*PO*) pairs (x-axis) and sibling (*SIB*) pairs (y-axis) across N=51 phenotypes (8 from GS:SFHS and 43 from UKB). Each panel shows different parameters: genetic variance (*G.variance*, left), shared environmental variance (*S.variance*, middle), and inverse of shared environmental decay rate (1/*S.decay*, right). Points represent individual phenotypes, with shapes indicating the dataset (triangle: GS:SFHS, circle: UKB). Colors represent the consistency of decay patterns between *PO* and *SIB* pairs: *fast* (red, shared environmental effects decay faster than genetic effects in both *PO* and *SIB*), *slow* (blue, shared environmental effects decay slower than genetic effects in both *PO* and *SIB*), *similar* (dark grey, shared environmental effects decay at similar rate with genetic effects in both *PO* and *SIB*), and *different* (light grey, inconsistent decay patterns between *PO* and *SIB*). The dashed diagonal line represents reference ($y = x$) line.

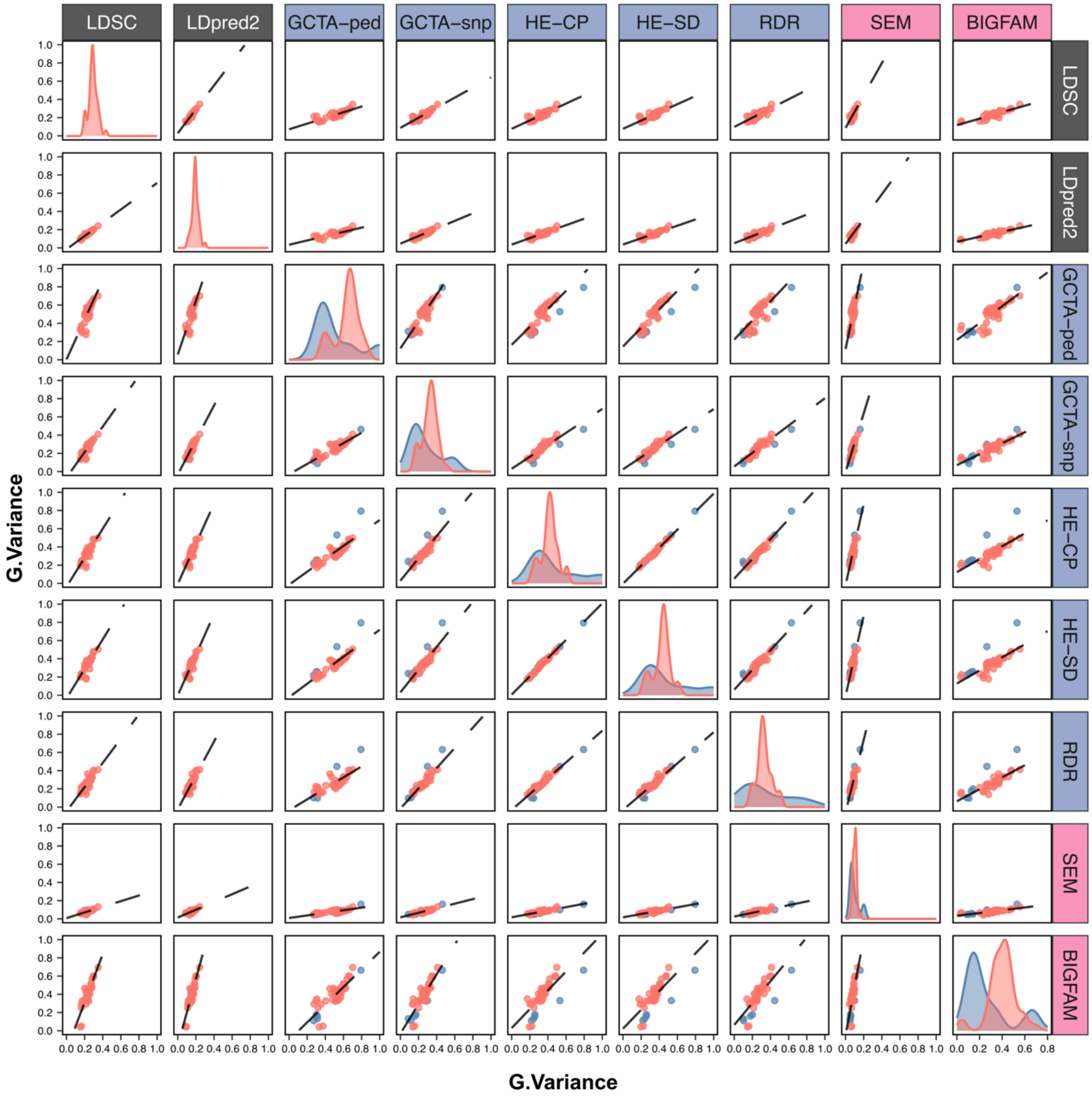

Figure 6. Pairwise comparison of genetic variance estimates across nine different methods.

Scatter plots show correlations between genetic variance (G.Variance) estimates from different methods based on N=40 phenotypes from UK Biobank (UKB) and Generation Scotland (GS:SFHS). Methods are grouped into three categories (shown in different background colors):

summary-based methods (white background: LDSC and LDpred2), genotype-based methods (blue background: GCTA-snp, GCTA-ped, HE-CP, HE-SD, RDR), and pedigree-based methods (pink background: BIGFAM, SEM). Methods within the same category show high correlation and concordance, particularly LDSC and LDpred2 demonstrating nearly perfect concordance, and HE-CP and HE-SD showing almost identical estimates. Red dots represent estimates from UK Biobank (UKB) dataset, and blue dots represent estimates from Generation Scotland: Scottish Family Health Study (GS:SFHS) dataset. Diagonal plots show the distribution of heritability estimates for each method, with red representing UKB and blue representing GS:SFHS. Black dashed lines represent regression line.

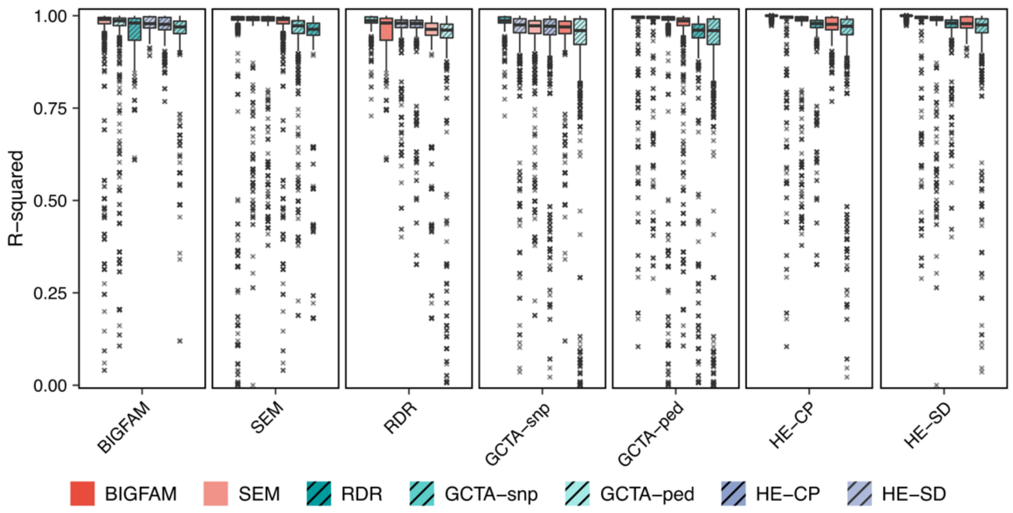

Figure 7. Pairwise Correlations Between Different Methods for Estimating Genetic Variance Components in GS:SFHS Dataset

Comparison of correlations (r^2) between seven methods for estimating genetic variance components in the GS:SFHS dataset, based on N=6 phenotypes that showed significantly non-zero genetic variance across all methods. Each panel shows correlations between the method indicated on x-axis and all other methods (shown by colored bars with different patterns). Methods are categorized into two groups: pedigree-based methods (BIGFAM, SEM; shown in solid red) and genotype-based methods (RDR, GCTA-snp, GCTA-ped, HE-CP, HE-SD; shown in teal with diagonal patterns). Error bars represent 95% confidence intervals calculated from 1,000 bootstrap resamples. Median correlations between methods were notably high ($r^2 >$ 0.95), the confidence intervals were substantially wider than in the UKB analysis due to the smaller number of phenotypes. Summary-based methods (LDSC and LDpred2) were not included in this analysis as the GS:SFHS dataset consists entirely of related individuals.

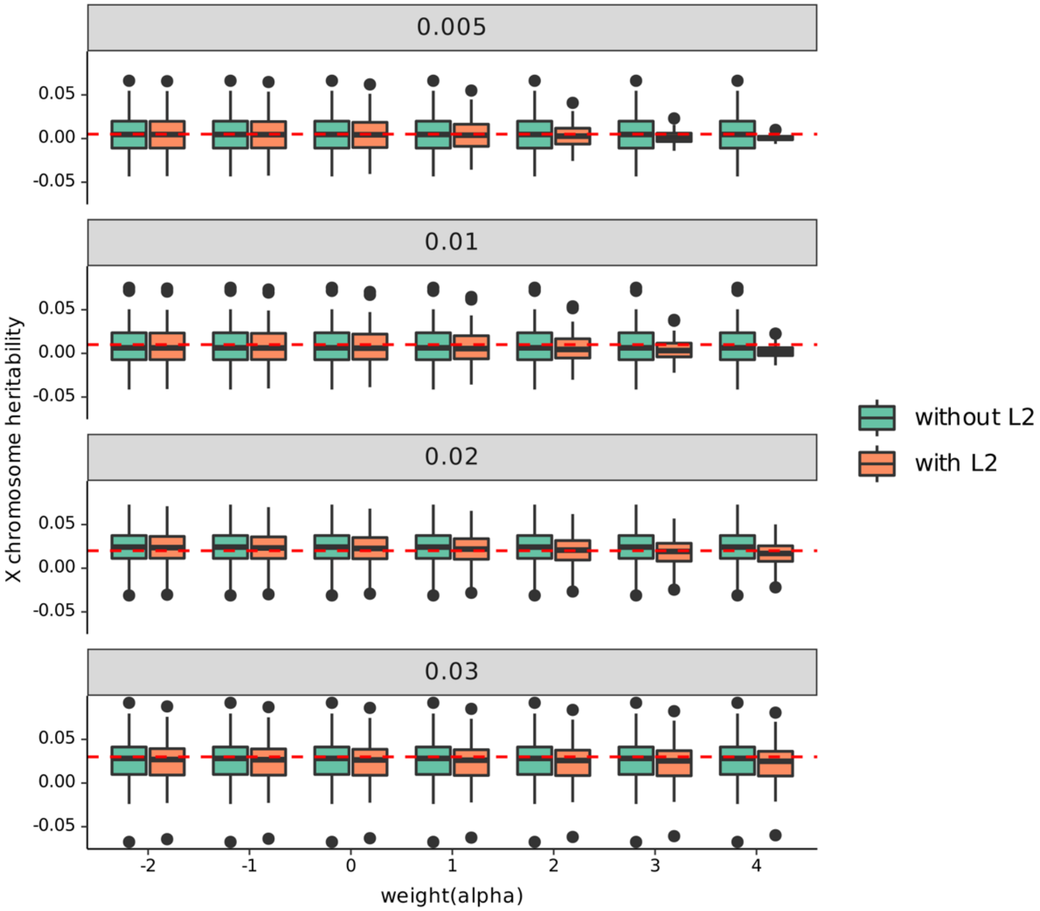

Figure 8. Effect of L2 Penalty Weight on X Chromosome Heritability Estimation

Comparison of X chromosome heritability estimates with and without L2 penalty across different penalty weights (α). Each panel represents different true X chromosome heritability (V_X) values (from top to bottom: 0.005, 0.01, 0.02, 0.03). Box plots show the distribution of estimated V_X from N=1,000 simulations per condition, with green boxes representing estimates without L2 penalty and orange boxes representing estimates with L2 penalty. The x-axis shows different L2 penalty weights (α) ranging from -2 to 4, and the y-axis shows the estimated V_X. Red dashed lines indicate true V_X. Black dots represent outliers.

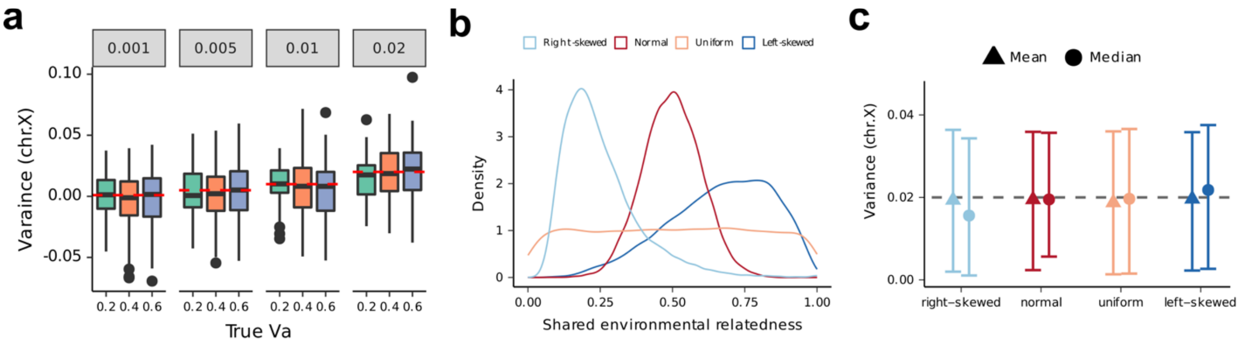

Figure 9. Performance Evaluation of BIGFAM's X Chromosome Variance Component Estimation in Simulation

a. Distribution of estimated X chromosome variance components (V_X) across different combinations of true autosomal (V_A: 0.2, 0.4, 0.6) and X chromosome variance (V_X: 0.001, 0.005, 0.01, 0.02; shown in grey boxes). Box plots show the distribution of estimated values from N=1,000 simulations per condition, with different colors representing different simulation settings. Red dashed lines indicate true V_X values.

b. Four different distributions of shared environmental relatedness used in simulation studies: right-skewed (higher proportion of relatives with weak shared environmental effects in same degree of relationship), normal, uniform, and left-skewed (higher proportion of relatives with strong shared environmental effects in same degree of relationship).

c. Performance evaluation under four different shared environmental correlation distributions with fixed true values ($V_A = 0.4$, $V_X = 0.02$). For each distribution, 1,000 simulations were performed, with each simulation providing a median estimate and its 95% confidence intervals. Triangles show the mean of 1,000 median estimates, with their error bars extending from the mean of lower bounds to the mean of upper bounds. Circles show the median of 1,000 median estimates, with their error bars extending from the median of lower bounds to the median of upper bounds. Black dashed line indicates the true V_X value (0.02).

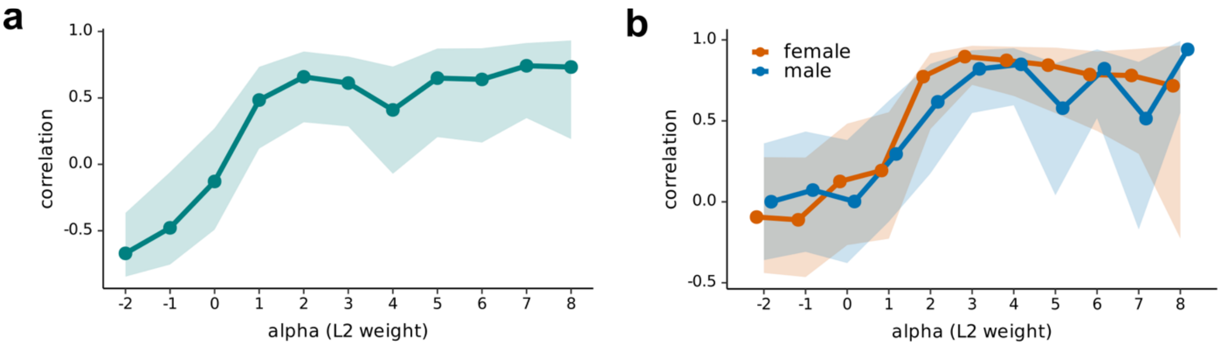

Figure 10. Correlation Between BIGFAM and SNP heritability on X Chromosome Across Different L2 Weights

a. Correlation of overall X chromosome heritability estimates between BIGFAM and unrelated samples using genotype data (SNP heritability) based on N=21 phenotypes. Shaded areas represent 95% confidence intervals obtained from 1,000 bootstrap resamples. **b**. Sex-stratified correlations between BIGFAM and unrelated samples using genotype data (SNP heritability) for male (blue) and female (orange) X chromosome heritability estimates based on N=24 phenotypes with significantly nonzero estimates. Shaded areas represent 95% confidence intervals obtained from 1,000 bootstrap resamples.

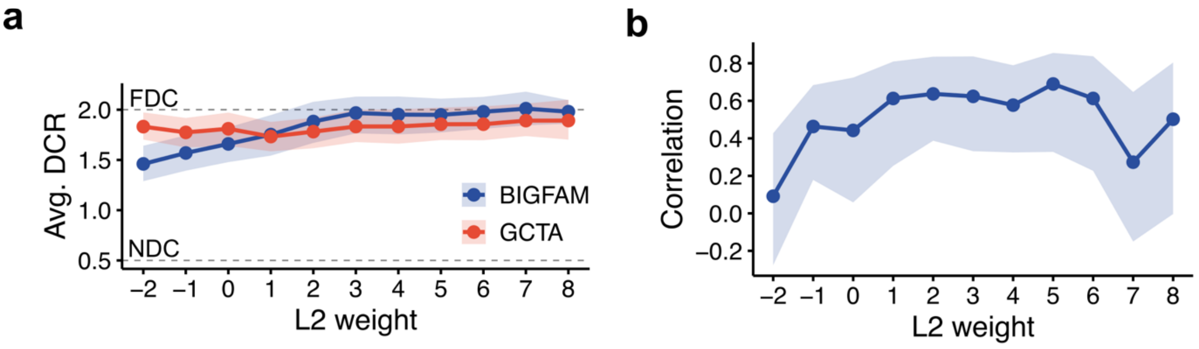

Figure 11. Comparison of DCR Estimates Between BIGFAM and Unrelated Samples Across Different L2 Weights

a. Average DCR values estimated by BIGFAM (blue) and from unrelated samples using GCTA (orange) across different L2 weights based on N=24 phenotypes with significantly nonzero sex-specific variance component by X chromosome. Dashed lines indicate theoretical expectations under full dosage compensation (FDC=2.0) and no dosage compensation (NDC=0.5). Both methods show consistent DCR estimates close to FDC across different L2 weights. **b**. Correlation between DCR estimates from BIGFAM and unrelated samples for the same N=24 phenotypes. Shaded areas in both panels represent 95% confidence intervals obtained from n=1,000 bootstrap resamples.

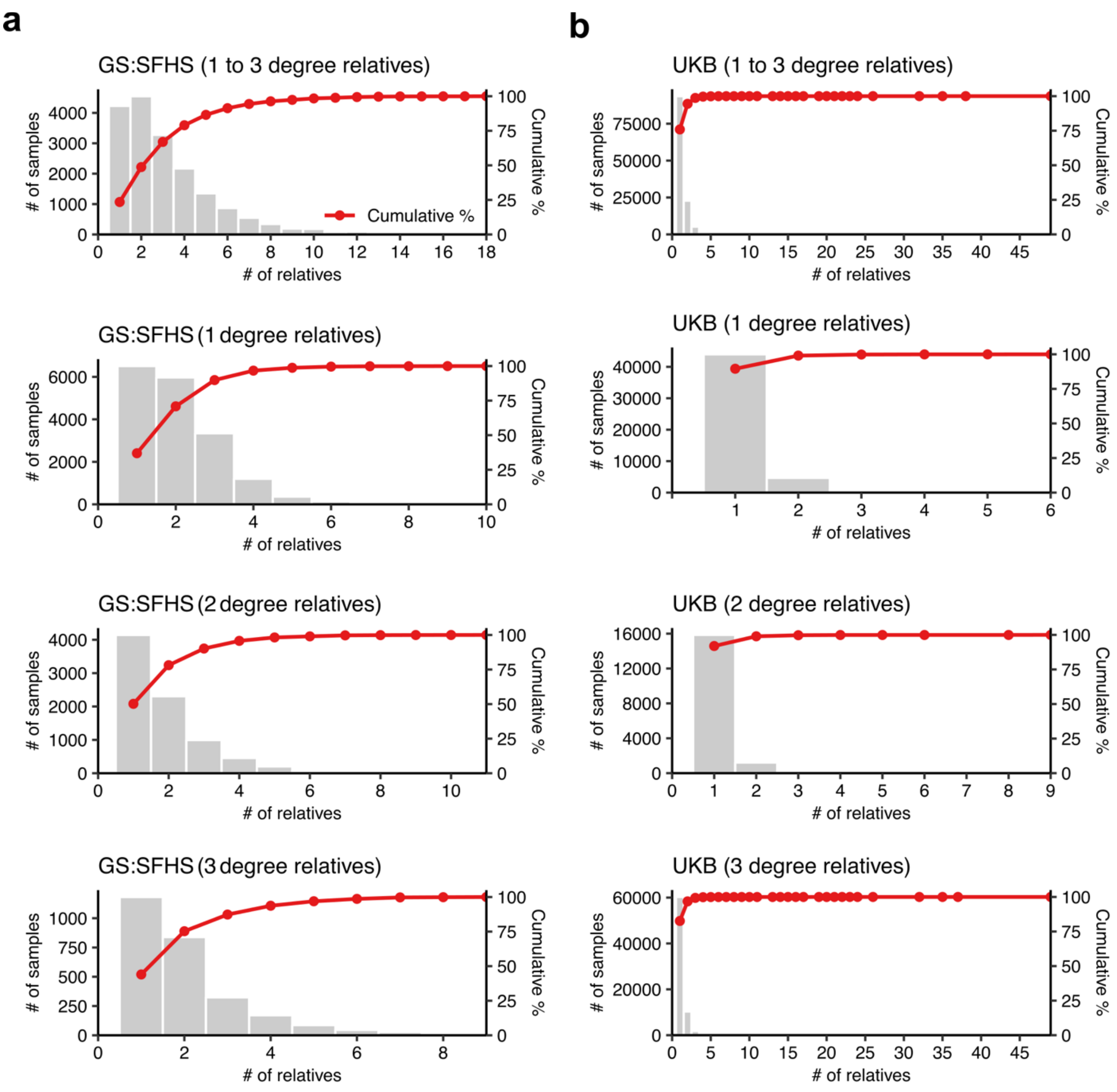

Figure 12. Distribution of the number of relatives per individual in GS:SFHS and UKB datasets.

a. Distribution of the number of relatives per individual in the GS:SFHS dataset (N=18,236 individuals). Top panel shows the overall distribution across all degrees (1st to 3rd degree relatives), followed by separate distributions for individuals with 1st, 2nd, and 3rd degree relatives. Grey bars represent the number of individuals (left y-axis) having specific numbers of

relatives (x-axis). Red lines show the cumulative percentage (right y-axis). **b.** Distribution of the number of relatives per individual in the UKB dataset (N=123,418 individuals). Similar to GS:SFHS, the top panel shows the overall distribution (1st to 3rd degree relatives), followed by degree-specific distributions. Note the different scales on both axes compared to GS:SFHS, reflecting the larger sample size of UKB. In both datasets, most individuals have relatively few relatives, as shown by the steep initial rise in the cumulative percentage curves. The distributions differ between datasets, with UKB showing more concentrated patterns (most individuals having fewer relatives) compared to the more spread-out distributions in GS:SFHS.

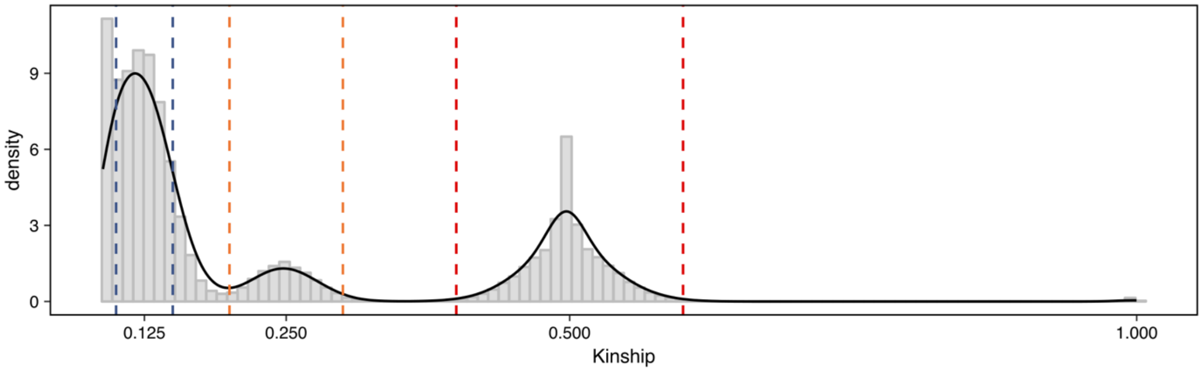

Figure 13. Distribution of kinship coefficients in UKB dataset. The density plot shows the distribution of marker-based kinship coefficients between N=81,326 pairs of individuals (123,418 individuals). Vertical dashed lines indicate the ranges used to infer degree-of-relatedness: first-degree relatives (red, 0.4-0.6), second-degree relatives (orange, 0.2-0.3), and third-degree relatives (blue, 0.10-0.15).

Supplementary Tables

DOR	Relation type	Relationship	N_pair	N_indiv	X correlation
1	all	all	27365	49083	
1	parent-child	daughter-father	949	1878	0.70
1	parent-child	daughter-mother	1874	3704	0.50
1	parent-child	son-father	637	1267	0.00
1	parent-child	son-mother	1281	2528	0.71
1	sibling	daughter-sister	3137	6102	0.75
1	sibling	different-sex-sibling	1861	3688	0.35
1	sibling	son-brother	665	1319	0.50
2	all	all	9447	17240	
2	avuncular	daughter-father-brother	286	565	0.35
2	avuncular	daughter-father-sister	330	652	0.25
2	avuncular	daughter-mother-brother	229	457	0.18
2	avuncular	daughter-mother-sister	579	1132	0.38
2	avuncular	son-father-brother	586	1143	0.00
2	avuncular	son-mother-brother	98	194	0.25
2	avuncular	son-mother-sister	346	683	0.53
3	all	all	44514	72944	
3	first-cousin	daughter-(father/mother)-brother-daughter	921	1810	0.25
3	first-cousin	daughter-father-sister-daughter	935	1816	0.13
3	first-cousin	daughter-mother-sister-daughter	1274	2484	0.19
3	first-cousin	son-(father-brother)/(father-sister)-daughter	5970	11393	0.00
3	first-cousin	son-(father-sister)/(father-sister)/(mother-brother)-son	3369	6408	0.00
3	first-cousin	son-mother-brother-daughter	947	1875	0.18
3	first-cousin	son-mother-sister-daughter	1051	2087	0.27
3	first-cousin	son-mother-sister-son	287	570	0.38

Table 1. Familial relationships inferred from UKB

DOR	Relation Type	Relationship	N_pair	N_indiv	X correlation
1	all	all	18258	17605	
1	parent-child	daughter-father	2147	3777	0.71
1	parent-child	daughter-mother	3506	5972	0.50
1	parent-child	son-father	1716	3087	0.00
1	parent-child	son-mother	2442	4411	0.71
1	sibling	daughter-sister	3180	4933	0.75
1	sibling	different-sex-sibling	3797	6185	0.35
1	sibling	son-brother	1470	2479	0.50
2	all	all	15114	8253	
2	avuncular	daughter-father-brother	1052	840	0.35
2	avuncular	daughter-father-sister	1440	1072	0.25
2	avuncular	daughter-mother-brother	1716	1343	0.18
2	avuncular	daughter-mother-sister	3206	2258	0.38
2	avuncular	son-father-brother	978	795	0.00
2	avuncular	son-father-sister	1178	919	0.00
2	avuncular	son-mother-brother	1406	1106	0.25
2	avuncular	son-mother-sister	2174	1581	0.53
2	grandparent	daughter-father-mother	196	177	0.50
2	grandparent	daughter-mother-father	200	172	0.35
2	grandparent	daughter-mother-mother	229	404	0.25
2	grandparent	son-father-mother	170	154	0.00
2	grandparent	son-mother-father	144	131	0.50
2	grandparent	son-mother-mother	372	328	0.35
2	half-sibling	daughter-mother-daughter	162	263	0.25
2	half-sibling	daughter-mother-son	149	260	0.35
2	half-sibling	son-mother-daughter	149	260	0.35
3	all	all	4634	2684	
3	first-cousin	daughter-father-brother-daughter	107	150	0.25
3	first-cousin	daughter-father-sister-daughter	594	433	0.12
3	first-cousin	daughter-mother-sister-daughter	396	512	0.19
3	first-cousin	son-father-brother-daughter	356	283	0.00
3	first-cousin	son-father-sister-daughter	490	382	0.00
3	first-cousin	son-father-sister-son	486	342	0.00
3	first-cousin	son-mother-brother-daughter	518	386	0.18
3	first-cousin	son-mother-sister-daughter	930	653	0.27
3	first-cousin	son-mother-sister-son	146	218	0.37
3	half-avuncular	son-mother-mother-daughter	194	160	0.18

Table 2. Familial relationships inferred from GS:SFHS

Supplementary Reference

1. Torvik, F. A. *et al.* Modeling assortative mating and genetic similarities between partners, siblings, and in-laws. *Nat Commun* **13**, 1108 (2022).

2. Hedrick, P. W. Assortative Mating and Linkage Disequilibrium. *G3 Genes|Genomes|Genetics* **7**, 55–62 (2017).

3. Border, R. *et al.* Assortative mating biases marker-based heritability estimators. *Nat Commun* **13**, 660 (2022).

4. Information (US), N. C. for B. *Genes and Disease*. (National Center for Biotechnology Information (US), 1998).

5. Bulik-Sullivan, B. K. *et al.* LD Score regression distinguishes confounding from polygenicity in genome-wide association studies. *Nat Genet* **47**, 291–295 (2015).

6. Privé, F., Albiñana, C., Arbel, J., Pasaniuc, B. & Vilhjálmsson, B. J. Inferring disease architecture and predictive ability with LDpred2-auto. *The American Journal of Human Genetics* **110**, 2042–2055 (2023).

7. Yang, J., Lee, S. H., Goddard, M. E. & Visscher, P. M. GCTA: A Tool for Genome-wide Complex Trait Analysis. *The American Journal of Human Genetics* **88**, 76–82 (2011).

8. Yang, J., Zeng, J., Goddard, M. E., Wray, N. R. & Visscher, P. M. Concepts, estimation and interpretation of SNP-based heritability. *Nat Genet* **49**, 1304–1310 (2017).

9. Zaitlen, N. *et al.* Using Extended Genealogy to Estimate Components of Heritability for 23 Quantitative and Dichotomous Traits. *PLOS Genetics* **9**, e1003520 (2013).

10. Young, A. I. *et al.* Relatedness disequilibrium regression estimates heritability without environmental bias. *Nat Genet* **50**, 1304–1310 (2018).

11. Butler, D. G., Cullis, B. R., Gilmour, A. R., Gogel, B. J. & Thompson, R. ASReml estimates variance components under a general linear.

12. Hill, W. D. *et al.* Genomic analysis of family data reveals additional genetic effects on intelligence and personality. *Mol Psychiatry* **23**, 2347–2362 (2018).
