# Supplementary material for: BIGFAM - variance components analysis from relatives without genotype
Source: Nat Commun. 2025 Jul 1;16:5476. doi: 10.1038/s41467-025-60502-0 (PMC12217023; doi:10.1038/s41467-025-60502-0)
Supplement: Supplementary file 2 — Reporting Summary [file 41467_2025_60502_MOESM2_ESM.pdf]

nature portfolio

Corresponding author(s): Buhm Han

Last updated by author(s): May 16, 2025

Reporting Summary

Nature Portfolio wishes to improve the reproducibility of the work that we publish. This form provides structure for consistency and transparency in reporting. For further information on Nature Portfolio policies, see our Editorial Policies and the Editorial Policy Checklist.

Statistics

For all statistical analyses, confirm that the following items are present in the figure legend, table legend, main text, or Methods section.

n/a	Confirmed	
☐	☒	The exact sample size (*n*) for each experimental group/condition, given as a discrete number and unit of measurement
☐	☒	A statement on whether measurements were taken from distinct samples or whether the same sample was measured repeatedly
☐	☒	The statistical test(s) used AND whether they are one- or two-sided<br>*Only common tests should be described solely by name; describe more complex techniques in the Methods section.*
☐	☒	A description of all covariates tested
☐	☒	A description of any assumptions or corrections, such as tests of normality and adjustment for multiple comparisons
☐	☒	A full description of the statistical parameters including central tendency (e.g. means) or other basic estimates (e.g. regression coefficient) AND variation (e.g. standard deviation) or associated estimates of uncertainty (e.g. confidence intervals)
☐	☒	For null hypothesis testing, the test statistic (e.g. *F*, *t*, *r*) with confidence intervals, effect sizes, degrees of freedom and *P* value noted<br>*Give P values as exact values whenever suitable.*
☒	☐	For Bayesian analysis, information on the choice of priors and Markov chain Monte Carlo settings
☒	☐	For hierarchical and complex designs, identification of the appropriate level for tests and full reporting of outcomes
☒	☐	Estimates of effect sizes (e.g. Cohen's *d*, Pearson's *r*), indicating how they were calculated

Our web collection on statistics for biologists contains articles on many of the points above.

Software and code

Policy information about availability of computer code

Data collection	No software was used to collect data.
Data analysis	Quality control pipeline for the genotype data was performed using PLINK v.1.90 and v.2.0. The heritability analysis performed using GCTA v.194, RDR (https://github.com/AlexTISYoung/RDR), and LDpred2 (bigsnpr v1.12). Results of LDSC is available at https://nealab.github.io/UKBB_ldsc. All BIGFAM code and analysis notebook is available at Github repository (https://github.com/jerrylee9310/BIGFAM)

For manuscripts utilizing custom algorithms or software that are central to the research but not yet described in published literature, software must be made available to editors and reviewers. We strongly encourage code deposition in a community repository (e.g. GitHub). See the Nature Portfolio guidelines for submitting code & software for further information.

Data

Policy information about availability of data

All manuscripts must include a data availability statement. This statement should provide the following information, where applicable:

- Accession codes, unique identifiers, or web links for publicly available datasets
- A description of any restrictions on data availability
- For clinical datasets or third party data, please ensure that the statement adheres to our policy

UK Biobank data is available at https://www.ukbiobank.ac.kr (project ID 285388) and Generation Scotland data is available at https://genscot.ed.ac.uk (GS23571). Public GWAS summary statistics used for LDSC are available at https://nealelab.github.io/UKBB_ldsc. Simulation inputs and all numeric values underlying the figures

are provided in the Source Data file accompanying this article. Custom code and processed results are deposited at GitHub (https://github.com/jerrylee9310/BIGFAM) and archived on Zenodo (DOI 10.5281/zenodo.15386299).

Research involving human participants, their data, or biological material

Policy information about studies with human participants or human data. See also policy information about sex, gender (identity/presentation), and sexual orientation and race, ethnicity and racism.

Reporting on sex and gender	The sex of individuals is used to infer familial relationships between relative pairs. Sex information is obtained from self-reported response and double-checked by the X chromosome abnormality.
Reporting on race, ethnicity, or other socially relevant groupings	Individuals who have only European ancestry were used in this analysis to control for potential confounding factors that may arise when multiple populations were used. Additionally, no socially relevant categorization variables were utilized in this analysis.
Population characteristics	Age an sex were used as covariates.<br>- UK Biobank (n=123,418 after QC): age=39–73y, mean(sd)=57.0(8.1)y; 56% female, 44% male.<br>- Generation Scotland: (n=18,236 after QC): age=18–99y, mean(sd)=46.5(15.3)y; 59% female, 41% male.
Recruitment	Participants in UKB and GS:SFHS were recruited from the overall cohort dataset.
Ethics oversight	The study protocol was approved by the Institutional Review Board (IRB) at Seoul National University Hospital Biomedical Research Institute (IRB#. E-2302-003-1400), UK Biobank (Project ID 285388), and Generation Scotland (GS23571).

Note that full information on the approval of the study protocol must also be provided in the manuscript.

Field-specific reporting

Please select the one below that is the best fit for your research. If you are not sure, read the appropriate sections before making your selection.

☒ Life sciences ☐ Behavioural & social sciences ☐ Ecological, evolutionary & environmental sciences

For a reference copy of the document with all sections, see nature.com/documents/nr-reporting-summary-flat.pdf

Life sciences study design

All studies must disclose on these points even when the disclosure is negative.

Sample size	We included all first- to third-degree relative pairs available after standard quality control: 81,326 pairs (123,418 individuals) in UK Biobank and 40,254 pairs (18,236 individuals) in Generation Scotland. Using the complete related-pairs sets maximises precision and avoids selection bias.
Data exclusions	From UKB, the relative individuals who have non-EUR ancestry, abnormal sex chromosome, inconsistent gender response, and inappropriate age difference to specify the familial relationship were excluded in this study.
Replication	We independently applied BIGFAM to two cohorts (UKBiobank and Generation Scotland) and to multiple simulation scenarios; in every case the method produced stable estimates that were internally consistent and, where comparable, concordant with genotype-based benchmarks.
Randomization	No experimental group allocation was performed. The study is retrospective and purely observational: all analyses use pre-existing UK Biobank and Generation Scotland data, treating each pair of related individuals as an observational unit. Since there is no intervention or comparison of assigned groups, randomization is not applicable. Potential confounders (age, sex) were controlled by regressing them out of the phenotypes before variance-component analysis.
Blinding	Blinding is not applicable to this study. All analyses were performed on previously collected UK Biobank and Generation Scotland datasets using inferred and pre-existing family-relationship labels; no experimental group assignment or subjective outcome scoring occurred.

Reporting for specific materials, systems and methods

We require information from authors about some types of materials, experimental systems and methods used in many studies. Here, indicate whether each material, system or method listed is relevant to your study. If you are not sure if a list item applies to your research, read the appropriate section before selecting a response.

Materials & experimental systems

n/a	Involved in the study
☒	☐ Antibodies
☒	☐ Eukaryotic cell lines
☒	☐ Palaeontology and archaeology
☒	☐ Animals and other organisms
☒	☐ Clinical data
☒	☐ Dual use research of concern
☒	☐ Plants

Methods

n/a	Involved in the study
☒	☐ ChIP-seq
☒	☐ Flow cytometry
☒	☐ MRI-based neuroimaging

Plants

Seed stocks

Report on the source of all seed stocks or other plant material used. If applicable, state the seed stock centre and catalogue number. If plant specimens were collected from the field, describe the collection location, date and sampling procedures.

Novel plant genotypes

Describe the methods by which all novel plant genotypes were produced. This includes those generated by transgenic approaches, gene editing, chemical/radiation-based mutagenesis and hybridization. For transgenic lines, describe the transformation method, the number of independent lines analyzed and the generation upon which experiments were performed. For gene-edited lines, describe the editor used, the endogenous sequence targeted for editing, the targeting guide RNA sequence (if applicable) and how the editor was applied.

Authentication

Describe any authentication procedures for each seed stock used or novel genotype generated. Describe any experiments used to assess the effect of a mutation and, where applicable, how potential secondary effects (e.g. second site T-DNA insertions, mosiacism, off-target gene editing) were examined.
